# Decoding the immune microenvironment: precision immunotherapy for medulloblastoma subtypes

**DOI:** 10.3389/fimmu.2026.1714134

**Published:** 2026-04-13

**Authors:** Mengyuan Li, Jihong Peng, Chun Chen, Jinyang Hu

**Affiliations:** 1 Department of Anesthesiology, The First College of Clinical Medical Science, Three Gorges University and Yichang Central People’s Hospital, Yichang, China; 2 Center for Medical Ultrasound, The Affiliated Suzhou Hospital of Nanjing Medical University, Suzhou Municipal Hospital, Suzhou, China; 3 Wenzhou Medical University, Wenzhou, China; 4 Department of Neurosurgery, The Second Affiliated Hospital of Wenzhou Medical University, Wenzhou, China

**Keywords:** CAR-T cell therapy, immune checkpoint blockade, immunotherapy, medulloblastoma subtypes, tumor immune microenvironment

## Abstract

Medulloblastoma is a severe pediatric brain tumor with distinct molecular subtypes—WNT, SHH, Group 3, and Group 4-each having unique genetic drivers and immune microenvironments. This review highlights the immune characteristics of each subtype: SHH is rich in tumor-associated macrophages (TAMs), whose role in tumorigenesis is debated; Group 3 features cytotoxic T cells often neutralized by immune checkpoints like PD-L1, causing T cell exhaustion; and Group 4 is marked by natural killer (NK) cells and B cells. These immune landscapes, including tumor-associated astrocytes (TAAs) and abnormal vascular networks, influence tumor growth, spread, and treatment response. Precision immunotherapy must be tailored to specific subtypes. This article discusses CAR T-cell therapy targeting antigens like B7-H3 and GD2, prevalent in SHH subtypes, and examines immune checkpoint blockades targeting PD-1/PD-L1 and CD47–SIRPα. It also highlights innovative methods like oncolytic viruses to transform “cold” tumor microenvironments and combination therapies using CSF1R inhibitors and tumor-associated antigens to boost anti-tumor responses. Understanding the immune microenvironment’s subtype-specific heterogeneity in medulloblastoma is crucial for advancing precision immunotherapy and improving patient outcomes.

## 1 Introduction

Medulloblastoma (MB), the most prevalent malignant embryonal tumor of the central nervous system in children, accounting for approximately 20% of all pediatric brain tumors (1, 2), is experiencing a paradigm shift in therapeutic strategy from histology-based classification toward molecular subtype-guided precision treatment (3, 4). Through comprehensive multi-omics analyses of extensive patient cohorts, four consensus molecular subgroups have been delineated: WNT-activated, SHH-activated, Group 3, and Group 4 (5). These subgroups demonstrate considerable heterogeneity in clinical presentation, tumor microenvironment composition, and treatment response (6). The World Health Organization’s classification system has incorporated these molecular characteristics alongside traditional histological assessments, emphasizing the pivotal role of molecular subtyping in clinical decision-making (7). Although current multimodal treatment strategies have significantly improved patient survival over the past few decades, approximately 30% of patients still have a dismal prognosis and ultimately succumb to the disease due to recurrence and metastasis (8). The long-term sequelae associated with surgery, radiotherapy, and chemotherapy-such as neurocognitive deficits and endocrine dysfunctions substantially impair the quality of life of survivors (9, 10). This underscores the limitations of existing therapies and the pressing need for more targeted and less toxic treatment modalities.

The tumor immune microenvironment (TME), a multifaceted ecosystem comprising immune cells, stromal cells, cytokines, and the extracellular matrix, is pivotal in tumor initiation, progression, metastasis, and therapeutic resistance (11–13). Recently, the role of the immune system in regulating MB has garnered increased scholarly attention. Traditionally, MB has been regarded as an “immune desert” or “immunologically cold tumor” in comparison to other brain tumors such as glioblastoma, primarily due to the presence of the blood-brain barrier and its relatively low tumor mutational burden (14, 15). However, recent molecular subtyping studies have contested this traditional perspective. Emerging evidence suggests that the TME of MB is not homogeneous but instead exhibits significant subtype-specific heterogeneity, which substantially affects clinical outcomes and therapeutic approaches (16). Although the WNT subtype is associated with the most favorable prognosis, it is characterized by notably sparse immune cell infiltration and low immunogenicity. The Sonic Hedgehog (SHH) subtype is characterized by an enrichment of TAMs, though the functional polarization of these cells remains a subject of debate (17). Some studies propose that TAMs expressing colony-stimulating factor 1 receptor (CSF1R) facilitate tumor progression (18), whereas other research suggests that certain TAM subsets may have tumor-suppressive roles (19). The Group 3 subtype, despite its infiltration by CD8+ T cells, is often linked to upregulation of programmed death-ligand 1 (PD-L1) and subsequent T cell exhaustion (16), with cyclin-dependent kinase 5 (CDK5)-mediated immune checkpoint activation identified as a pivotal mechanism (20). Conversely, the Group 4 subtype is distinctively marked by substantial infiltration of natural killer (NK) cells and B cells, the latter of which is associated with improved prognosis (21). The immune environment of each subtype is shaped by genetic changes, distinct cytokine networks like CCL2-driven monocyte recruitment in SHH subtypes, unusual angiogenesis, and interactions with tumor-associated astrocytes (19, 22).

Understanding the heterogeneity of the immune microenvironment is essential for the development of precision immunotherapy strategies tailored to specific subtypes. This encompasses the rational selection of immune checkpoint inhibitors, the design of antigen-specific CAR-T cell therapies, and the formulation of combination strategies aimed at reprogramming TAMs. By systematically dissecting the cellular composition, spatial architecture, and signaling network features of the immune microenvironment across molecular subtypes, this review seeks to provide a conceptual framework and translational roadmap for advancing precision immunotherapy in medulloblastoma.

## 2 The molecular blueprint of medulloblastoma

**Figure 1:**
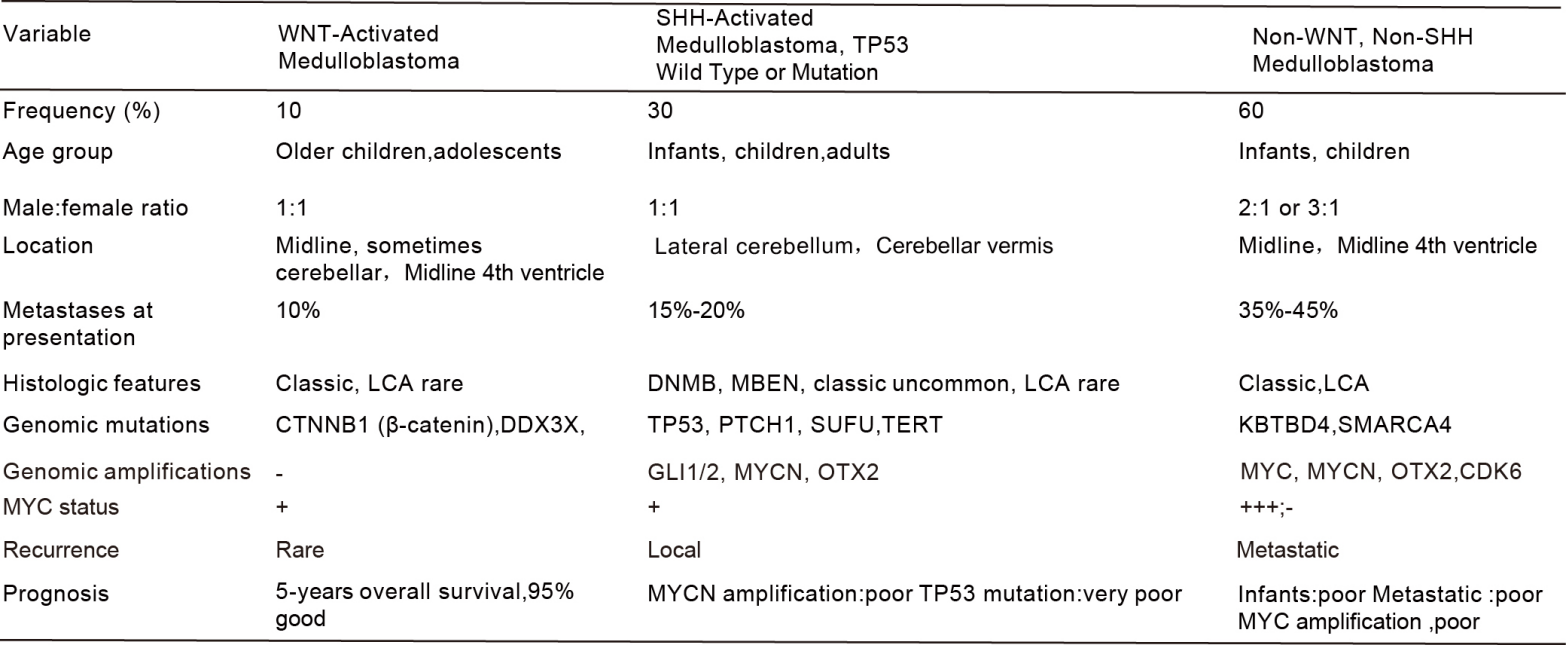
The molecular blueprint of medulloblastoma based on 2021 World Health Organization classification of central nervous system tumors. WNT, Wingless; SHH, Sonic-hedgehog; MYC, Myelocytomatosis oncogene, encoding a transcription factor critical for cell proliferation; DNMB, denotes desmoplastic nodular medulloblastoma; LCA, large-cell anaplastic, and MBEN, medulloblastoma with extensive nodularity.

The World Health Organization (WHO) classification of central nervous system tumors has integrated molecular stratification with histopathology, providing a more precise framework for clinical diagnosis and risk stratification. This section systematically reviews the key features of each subtype, whose heterogeneity lays the groundwork for understanding differences in the TME discussed subsequently (**Figure 1**).

### 2.1 WNT-activated medulloblastomas

The WNT subtype of medulloblastoma is associated with the most favorable prognosis among its subgroups, comprising approximately 10% of all cases (23). This subtype predominantly affects older children and adolescents, with infrequent occurrence in infants. On a molecular level, it is characterized by the aberrant activation of the WNT signaling pathway, with somatic catenin beta 1 (CTNNB1) mutations identified in about 90% of cases (24). These mutations are often accompanied by monosomy 6 and other genetic alterations, such as mutations in DEAD-box helicase 3 X-linked (DDX3X) and SMARCA4 (1). Tumors are typically situated in the fourth ventricle, frequently involving the brainstem, and exhibit classic histological features (25). The exceptional prognosis of this subtype is largely attributed to the mutant β-catenin-driven aberrant vascular fenestration, which disrupts the blood-brain barrier and significantly enhances the penetration of chemotherapeutic agents (26). Even in cases with metastasis, the 5-year survival rate exceeds 90%, and the 10-year event-free survival rate surpasses 95% (27). Considering these outstanding outcomes, current clinical research is focused on de-escalation strategies aimed at reducing the intensity of radiotherapy and chemotherapy.

### 2.2 SHH-activated medulloblastomas

The Sonic Hedgehog (SHH)-activated medulloblastoma subtype constitutes approximately 30% of all medulloblastoma cases and is characterized by a distinct bimodal age distribution, predominantly affecting infants and adults, while being relatively rare in children (6). This subtype exhibits an equal sex distribution and typically originates in the cerebellar hemispheres from precursor cells in the external granular layer (28, 29). Histologically, the desmoplastic/nodular variant is most observed (30).

The pathogenesis of this subgroup is driven by constitutive activation of the Sonic Hedgehog signaling pathway, with recurrent genetic alterations such as patched 1 (PTCH1) mutations (43%), suppressor of fused homolog (SUFU) mutations (often associated with Gorlin syndrome), smoothened, frizzled class receptor (SMO) mutations (9%), and telomerase reverse transcriptase (TERT) promoter mutations (27). The tumor protein p53 (TP53) mutation status is the most critical prognostic factor: cases with wild-type TP53 generally have relatively favorable outcomes, whereas cases with TP53 mutations (9%) are highly aggressive (31), often exhibiting GLI family zinc finger 2 (GLI2) amplifications, and are associated with a poor prognosis (32). Based on these molecular characteristics, SMO inhibitors, such as vismodegib and sonidegib (33, 34), have been employed in the targeted therapy of refractory cases.

### 2.3 Non-WNT, non-SHH medulloblastomas

Non-WNT/non-SHH medulloblastomas encompass the Group 3 and Group 4 subtypes, both of which exhibit a marked male predominance and frequently present with metastases at the time of diagnosis (35). Group 3 tumors, accounting for approximately 25% of cases, predominantly affect infants and are characterized by MYC amplification (36), resulting in the poorest prognosis, with a 5-year overall survival rate of merely 50%. In contrast (37, 38), Group 4 tumors, which constitute about 35% of cases, are more prevalent in older children and are associated with the presence of isochromosome 17q (i17q) in 80% of instances, leading to an intermediate prognosis with a 5-year overall survival rate of 70% (39). Molecular stratification further categorizes Group 4 into high-risk and low-risk subgroups, with 10-year survival rates of 36% and 72%, respectively (40).

Current therapeutic approaches are tailored according to molecular classification: infants with Group 3 tumors receive high-dose chemotherapy accompanied by autologous stem cell rescue to postpone radiotherapy (41), whereas Group 4 patients are treated with risk-adapted regimens (42). Ongoing research endeavors aim to develop novel targeted therapies for these subtypes, as their driving mutations remain largely unidentified.

## 3 The immune microenvironment: a subtype-specific focus

The TME is a critical determinant of MB progression and therapeutic response. However, the MB TME is not a monolithic entity; it exhibits profound heterogeneity across the four core molecular subtypes. This section provides a detailed examination of the distinct immune landscapes that characterize the WNT, SHH, Group 3, and Group 4 subtypes, highlighting how these unique cellular compositions and interactions influence tumor biology and shape opportunities for immunotherapy.

### 3.1 The WNT subtype: a unique immune-privileged entity

Numerous studies have consistently indicated that the WNT subtype is characterized by significantly reduced levels of tumor-infiltrating lymphocytes (TILs), TAMs, dendritic cells (DCs), and other immune cells when compared to other subtypes, notably the SHH and Group 3 subtypes (16, 43). For example, a transcriptomic analysis conducted by Bockmayr demonstrated that the WNT subtype exhibits the lowest expression levels of immune-related genes, suggesting a limited presence and functional activity of immune cells within its TME (17, 44). Additionally, immune checkpoint molecules such as PD-L1 are typically low or even absent in the WNT subtype, further corroborating its “immune-quiet” phenotype (45–47). The correlation between this “immune-quiet” status and the favorable prognosis of the WNT subtype prompts a critical inquiry: Is this immune microenvironment the cause of the WNT subtype’s favorable prognosis, or is it a consequence of its inherently benign biological characteristics?

On one hand, some scholars posit that the “immune-quiet” nature may originate from the intrinsic biological attributes of the WNT subtype (45). The activation of the WNT signaling pathway may indirectly inhibit the recruitment or activation of immune cells by modulating processes such as cell adhesion, proliferation, and differentiation (48). For instance, WNT signaling can influence the expression of major histocompatibility complex (MHC) class I molecules on tumor cells or alter cytokine secretion, thereby diminishing T cell recognition and attack (49). Furthermore, WNT subtype tumors are typically situated in the midline of the fourth ventricle, where the blood-brain barrier remains relatively intact, potentially restricting the infiltration of peripheral immune cells (50).

On the other hand, some studies propose that the “immune-quiet” phenotype may be a consequence, rather than a cause, of the low invasiveness and metastatic potential of WNT tumors (51). Tumor cells of the WNT subtype exhibit relatively orderly proliferation (52), low mutational burden, and limited neoantigen expression, which may not sufficiently provoke a robust immune response (53). In essence, the immune system may not be adequately “alerted” because the tumor does not provide sufficient “danger signals.”.

**Figure 2:**
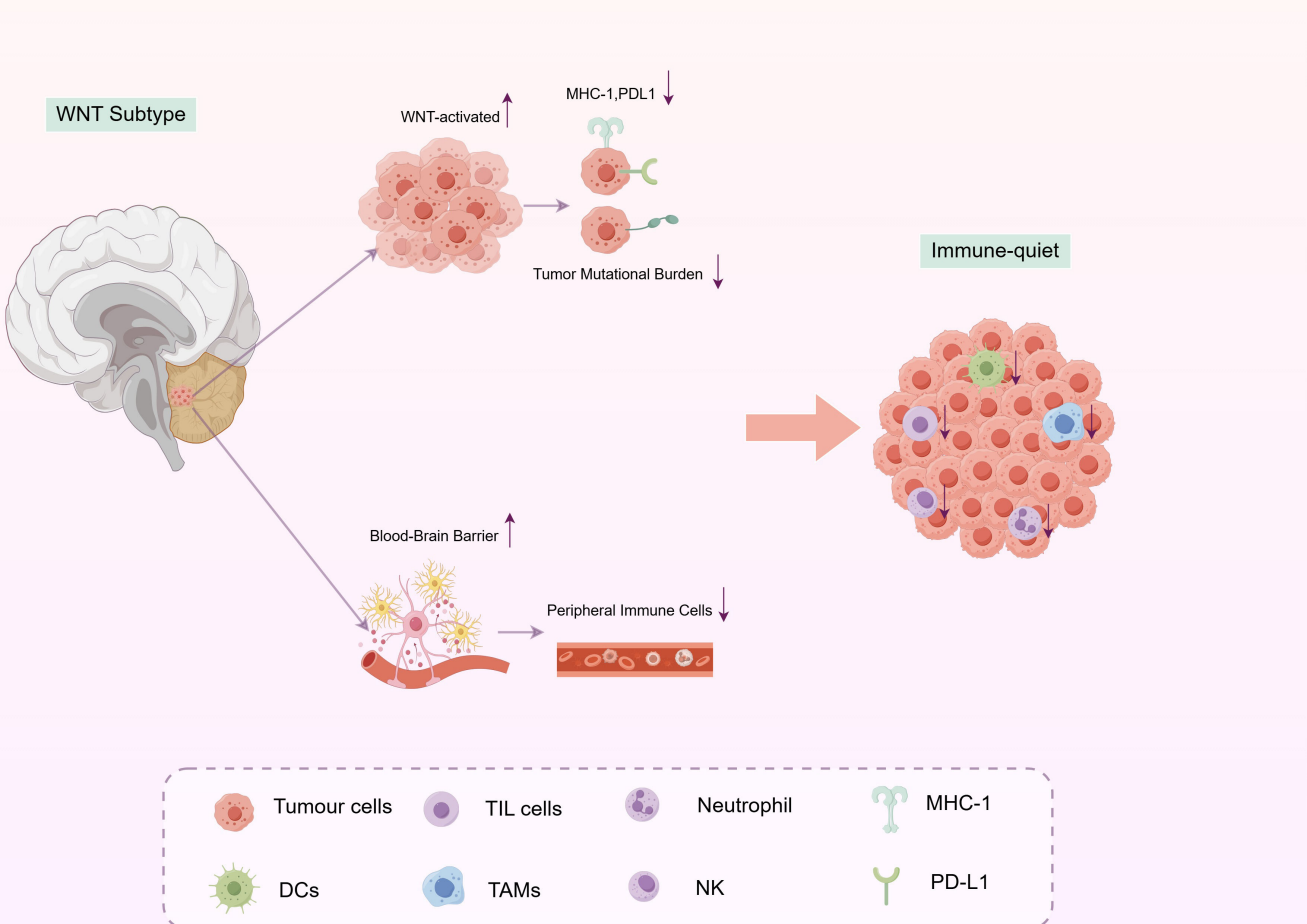
The “immune-quiet” tumor microenvironment of WNT-activated medulloblastoma. The WNT subtype of medulloblastoma exhibits a notably sparse immune microenvironment, characterized by low levels of infiltration of immune cells such as dendritic cells (DCs), tumor-infiltrating lymphocytes (TILs), tumor-associated macrophages (TAMs), neutrophils, and natural killer (NK) cells. This subtype also demonstrates low expression of MHC class I molecules and PD-L1, along with a low tumor mutational burden. The “immune-quiet” phenotype may stem from two main factors: intrinsic biological features—such as WNT pathway-mediated modulation of antigen presentation and cytokine secretion—and anatomical factors, including a relatively intact blood-brain barrier that restricts the entry of peripheral immune cells. This state of immune ignorance, distinct from active immune escape, may contribute to the favorable clinical prognosis associated with the WNT subtype.

It is important to clarify that the “immune-quiet” characteristic of the WNT subtype should not be misconstrued as “immune escape.” The latter term generally describes active mechanisms utilized by tumors to suppress immune responses, such as the upregulation of PD-L1 or the recruitment of regulatory T cells (Tregs). In contrast, the WNT subtype typically demonstrates a state of “immune ignorance,” which may, in fact, prevent tissue damage resulting from excessive immune activation or inflammation-driven tumor progression (**Figure 2**).

### 3.2 The SHH subtype: an immunologically “warmer” niche

**Figure 3:**
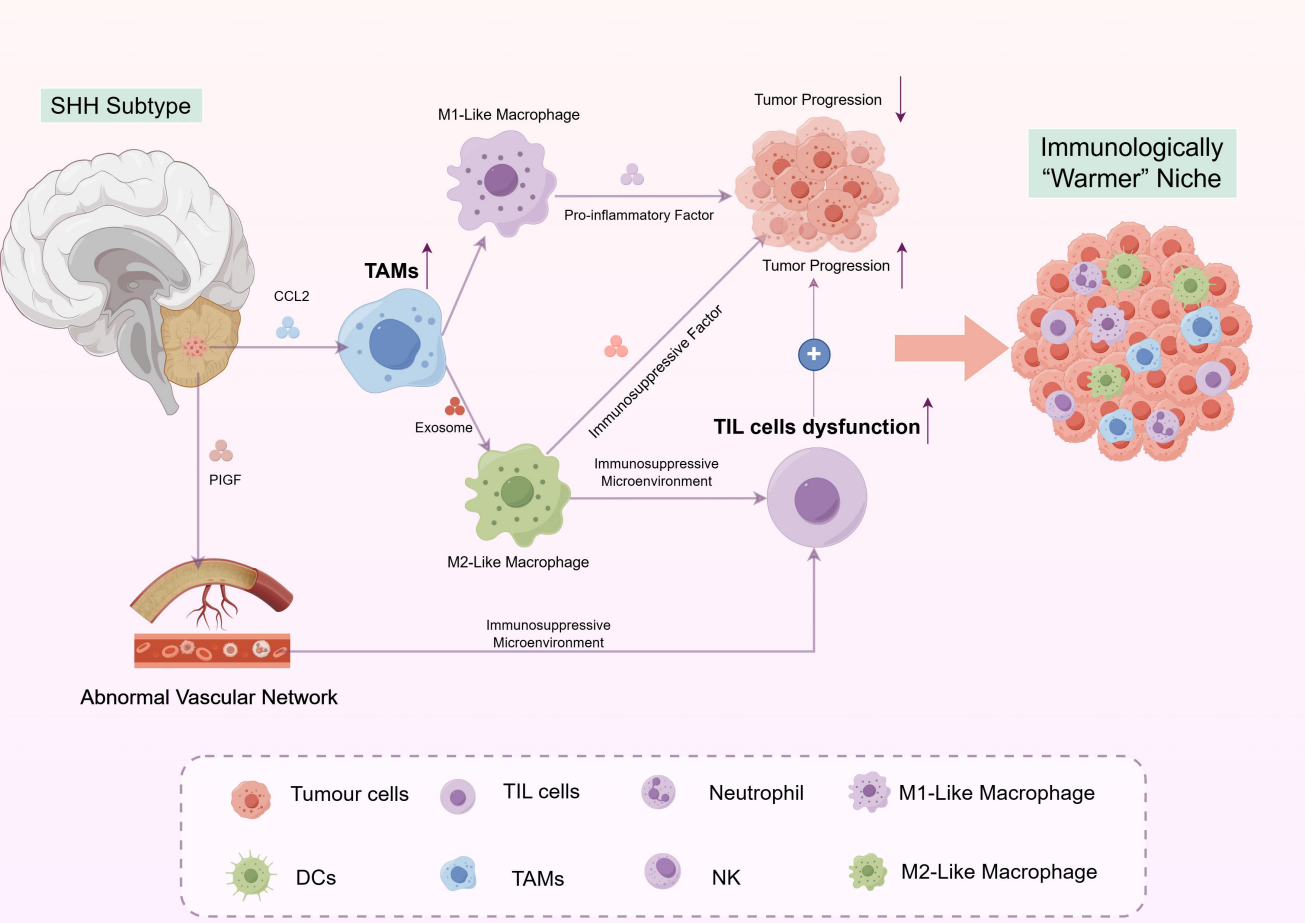
The complex and immunologically “warmer” tumor microenvironment of SHH-activated medulloblastoma. The SHH subtype is characterized by a highly cellular and vascularized tumor microenvironment, in which tumor-associated macrophages (TAMs) play a dominant role with dual functions. TAMs can polarize into an anti-tumor M1-like phenotype that induces tumor cell death, or into a pro-tumor M2-like state via exosomal microRNA-mediated PPARγ activation, thereby promoting immunosuppression and conferring resistance to SMO inhibitors. Although tumor-infiltrating lymphocytes (TILs) are present, their function is often suppressed within this immunosuppressive niche. An abnormal vascular network driven by PlGF/NRP-1 signaling supports tumor progression and metastasis. Key mediators such as CCL2 and exosomes regulate immune cell recruitment and polarization, collectively shaping a dynamic yet therapeutically targetable ecological microenvironment.

In stark contrast to the immune-quiescent WNT subtype, the SHH-activated medulloblastoma presents a more complex and cellularly rich TME. This subtype is distinguished by a prominent stromal reaction and a diverse immune infiltrate, positioning it as a prime candidate for immunotherapeutic interventions. However, the functional roles of these immune cells, particularly tumor-associated macrophages (TAMs), are multifaceted and context-dependent, creating a dynamic and often paradoxical microenvironment (**Figure 3**).

#### 3.2.1 The dominance and duality of tumor-associated macrophages

Extensive research consistently demonstrates that the SHH subtype of MB is characterized by a distinctive and immune-rich TME, in which TAMs play a predominant role (17, 18, 54, 55). Molecular analyses of patient samples have revealed a significant enrichment of TAM-associated genes and M2-like macrophage signatures within SHH tumors (56). Histopathological examination further corroborates the presence of high densities of CCR2^+^ infiltrating macrophages, derived from the peripheral circulation, in SHH tumors, in contrast to CX3C chemokine receptor 1 (CX3CR1)^+^ resident microglia (54). This suggests an active recruitment process mediated by tumor-derived factors, such as C-C motif chemokine ligand 2 (CCL2).

Contrary to the conventional perspective that TAMs are invariably pro-tumorigenic, seminal research by Maximov et al. has identified a protective role for TAMs in SHH-MB (19). Their study demonstrated that the reduction of TAMs through genetic knockout or pharmacological inhibition resulted in accelerated tumor progression, decreased apoptosis, and increased proliferation in murine SHH models. Furthermore, elevated expression of the macrophage marker AIF1 in human SHH patients was significantly associated with improved survival outcomes. Functional assays have demonstrated that bone marrow-derived macrophages (BMDMs) can induce the death of SHH tumor cells *in vitro*. This finding strongly indicates that, within SHH-MB, at least a subset of TAMs possesses an inherent anti-tumor capability, and their presence serves as a favorable prognostic marker.

However, this protective role is not absolute, and substantial evidence also supports the traditional pro-tumorigenic functions of TAMs in other contexts. Research conducted by Zhu et al. elucidated a mechanistic basis for this pro-tumor polarization in SHH-MB (57). They identified that SHH tumor cells release exosomes containing specific microRNAs, which, upon uptake by TAMs, mitigate the repression of Peroxisome Proliferator-Activated Receptor Gamma (PPARγ). This process drives macrophages towards an M2-like, pro-tumorigenic phenotype. Such polarization not only fosters immunosuppression but also contributes significantly to resistance against SMO inhibitors. The therapeutic implications of this discovery are underscored by the finding that the PPARγ antagonist GW9662 can reverse M2 polarization and work synergistically with SMO inhibition to effectively suppress tumor growth *in vivo*.

TAMs are prevalent in SHH MB In addition to TAMs and exhibit a dual role: they can either combat or promote tumor growth. This apparent contradiction underscores a fundamental limitation in our current understanding: we likely lack the tools to distinguish functionally distinct TAM subpopulations within the tumor bulk. The protective role observed by Maximov et al. may be attributable to a specific, and perhaps minor, subset of TAMs that is lost in the gross depletion strategies. Conversely, the pro-tumor effects may be driven by a different, potentially dominant, subset. This ambiguity has direct clinical implications. The failure of broad CSF1R inhibition strategies in some solid tumors serves as a cautionary tale; indiscriminate TAM depletion could inadvertently eliminate anti-tumor subsets, potentially yielding poor clinical outcomes. Therefore, the central challenge is no longer simply quantifying TAM abundance but rather defining the molecular determinants of their polarization and functional heterogeneity. Future therapeutic strategies must evolve from non-specific depletion towards precision modulation, such as selectively repolarizing M2-like TAMs or harnessing the cytotoxic potential of the protective subset identified by Maximov et al.

This inherent functional dichotomy is further complicated by the distinct origins and spatial distribution of macrophages within the TME. Tumor-associated macrophages in SHH-MB can originate from either resident microglia or bone marrow-derived monocytes recruited via the CCL2-CCR2 axis, as noted earlier (54). These ontogenically distinct populations are now understood to possess non-redundant functions (58). For instance, single-cell transcriptomic studies in other brain tumor contexts have revealed that microglia often maintain a more surveillance-like state, while monocyte-derived macrophages are more susceptible to adopting an immunosuppressive M2-like phenotype upon exposure to tumor-derived factors (57). The protective anti-tumor effects observed upon broad TAM depletion might therefore reflect the unintended elimination of tumor-suppressive microglia, overshadowing the pro-tumor effects of the potentially more abundant monocyte-derived population. This ontogenetic and functional heterogeneity is further amplified by spatial heterogeneity, as TAMs residing in distinct tumor niches, including the perivascular area, the invasive front, and the hypoxic core, are exposed to varying microenvironmental cues such as oxygen levels, metabolic substrates, and stromal cell interactions, which drive the polarization of discrete functional subpopulations (59, 60). A comprehensive understanding of SHH-MB immunity thus requires moving beyond a binary M1/M2 classification and mapping the full spectrum of TAM activation states in a spatially resolved manner.

Furthermore, the TAM landscape is not static but dynamically reshaped by therapeutic interventions. Conventional treatments like radiation and chemotherapy can induce significant macrophage reprogramming. For example, radiation therapy can elicit a potent anti-tumor immune response by promoting immunogenic cell death and attracting pro-inflammatory macrophages (61). Conversely, it can also trigger a wound-healing response, recruiting immunosuppressive TAMs that contribute to tumor recurrence (62). Similarly, SMO inhibitors, while targeting tumor cells, can alter the tumor secretome, thereby influencing the recruitment and polarization of TAMs. Treatment-induced TAM reprogramming may, in fact, underline the failure of some therapies, as a resistant and more aggressive tumor microenvironment is re-established.

This complexity provides a crucial context for understanding the limitations of therapeutic strategies aimed at broad TAM depletion, such as CSF1R inhibitors. While preclinical models showed initial promise by targeting the CSF1R-dependent survival of TAMs (63), their translation to the clinic has been largely disappointing in many solid tumors (64, 65). This failure can be attributed to several factors. First, targeting the wrong subset: indiscriminate CSF1R inhibition depletes both pro-tumor and anti-tumor TAM populations, potentially abrogating an endogenous source of tumor control (19). Second, compensatory mechanisms: the TME can adapt to CSF1R blockade by upregulating alternative survival pathways, such as IL-34 which also binds CSF1R, or other growth factors like GM-CSF, or by shifting the dependency of TAMs away from CSF1R, allowing a new wave of immunosuppressive macrophages to infiltrate (66, 67). Third, clinical trial design: many early trials were conducted in unselected patient populations, failing to account for the highly context- and subtype-specific roles of TAMs. A strategy that might be effective in a TAM-rich, macrophage-addicted tumor like SHH-MB could be ineffective in a T-cell inflamed tumor. In the specific case of SHH-MB, the protective role of certain TAM subsets identified by Maximov et al. serves as a powerful cautionary tale, suggesting that simply depleting all macrophages could be therapeutically detrimental. This underscores the critical need to move from non-specific depletion strategies toward precision modulation, such as selectively repolarizing pro-tumor M2-like TAMs, inhibiting their recruitment, or harnessing the cytotoxic potential of protective subsets rather than eliminating them entirely.

#### 3.2.2 Lymphocyte infiltration: presence amidst suppression

In addition to TAMs, the SHH medulloblastoma subtype is characterized by a relatively higher infiltration of T and B lymphocytes compared to non-SHH subgroups (17, 68). Nevertheless, this increased infiltration does not necessarily translate into effective anti-tumor immunity. The functional capacity of these lymphocytes is frequently compromised by the immunosuppressive environment orchestrated by TAMs and other stromal elements. The adaptive immune response is likely attenuated through mechanisms such as the upregulation of immune checkpoint molecules and the secretion of anti-inflammatory cytokines (57), thereby rendering the infiltrating lymphocytes ineffective in controlling tumor progression despite their presence.

#### 3.2.3 Vascular and stromal interactions: fueling progression and metastasis

The SHH tumor microenvironment is characterized by a highly vascularized stroma that not only supports tumor growth but also facilitates metastatic dissemination (69). A pivotal element in this process is the Placental Growth Factor (PlGF)/Neuropilin-1 (NRP-1) signaling pathway. The interaction between SHH-induced PlGF and NRP-1 within the cerebellar stroma has been identified as a crucial mechanism underlying medulloblastoma metastasis, which is associated with poorer patient prognoses (70). This aberrant vascular network not only supplies essential nutrients but also contributes to an immunosuppressive milieu by forming a physical barrier and expressing factors that inhibit effective immune cell function (71).

In summary, the SHH subtype presents an immunologically active yet complex niche, predominantly influenced by TAMs with ambiguous roles, accompanied by suppressed lymphocyte activity, and sustained by a pro-metastatic vascular system. This intricate interplay of interactions highlights the imperative for precisely targeted immunotherapies capable of modulating specific components of the SHH TME effectively.

### 3.3 Non-WNT, non-SHH Medulloblastomas: the “cold” but heterogeneous landscapes

**Figure 4:**
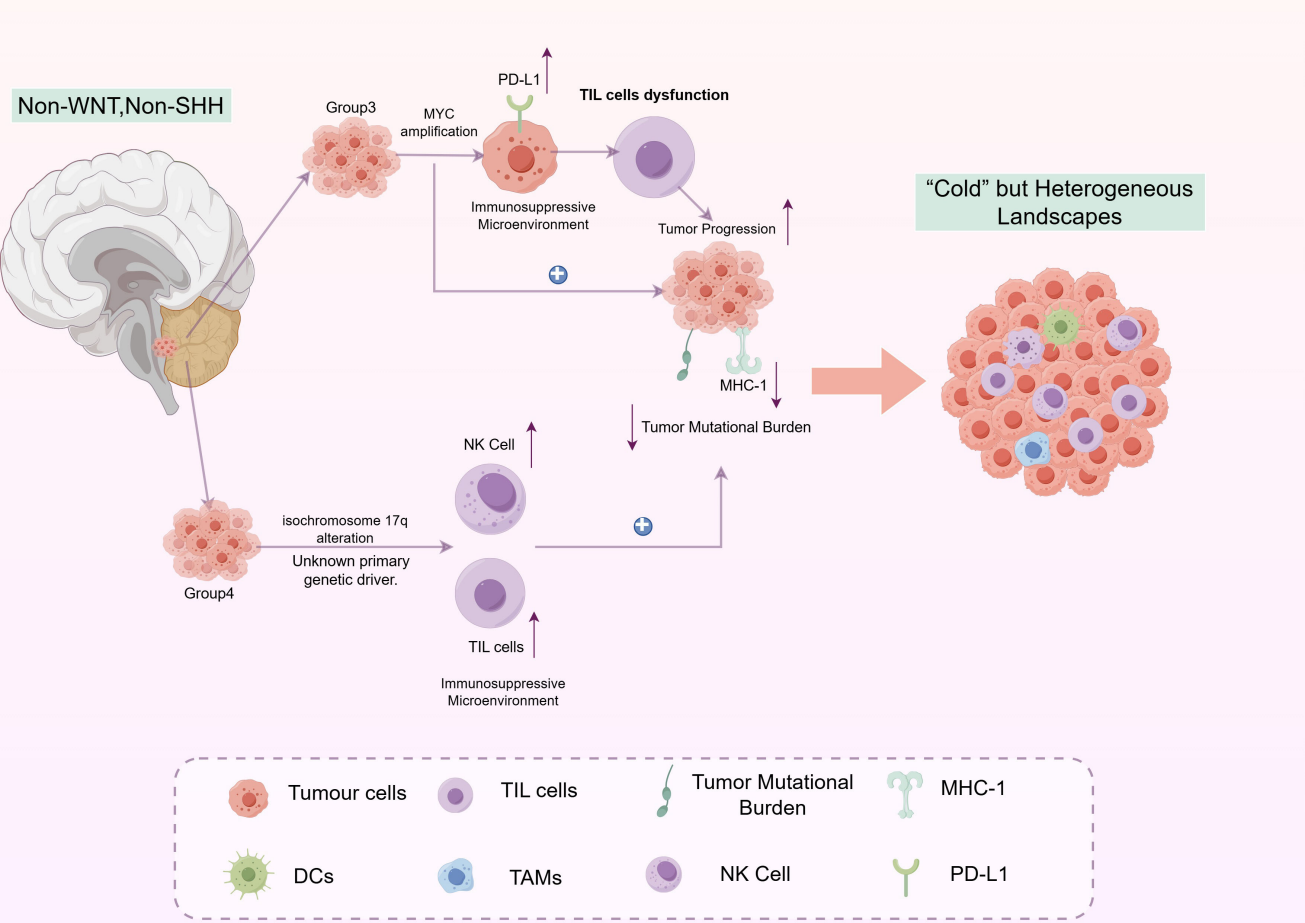
The heterogeneous yet immunologically “cold” tumor microenvironment (TME) of non-WNT/non-SHH medulloblastomas. Group 3 and Group 4 medulloblastomas share common features of a “cold” TME, including low tumor mutational burden and reduced MHC-I expression, which contribute to their low inherent immunogenicity. However, they exhibit distinct genetic drivers and immune contextures. The Group 3 subtype is defined by frequent MYC amplification, which drives an intensely immunosuppressive TME. MYC upregulates PD-L1 on tumor cells, leading to dysfunction and exhaustion of tumor-infiltrating lymphocytes (TILs), despite their presence. In contrast, the Group 4 subtype, often characterized by isochromosome 17q (i17q) alteration, displays a less aggressive TME. It features infiltration of innate immune cells like natural killer (NK) cells, and the presence of B cells is associated with a more favorable prognosis. The paradoxical presence of immune cells (TILs, TAMs, DCs) within these generally “cold” TMEs, coupled with dominant immunosuppressive mechanisms (particularly in Group 3), highlights the critical need for tailored immunotherapeutic strategies to overcome specific immune evasion pathways.

SHH and WNT medulloblastomas are driven by distinct signaling pathways, whereas Group 3 and Group 4 tumors are more complex and less defined. Previously categorized together as “non-WNT/non-SHH,” they are now seen as separate entities with common challenges but unique immune profiles. Both are immunologically “cold,” with low tumor mutational burden and reduced MHC-I expression, limiting their innate immunogenicity. Despite this, crucial differences beneath the surface are vital for developing therapies (**Figure 4**).

Group 3 MB represents the most clinically aggressive subtype, with approximately 50% of patients exhibiting metastatic disease at diagnosis. This unfavorable prognosis is intrinsically linked to the frequent amplification of the MYC oncogene. In addition to its role in promoting cellular proliferation (72, 73), MYC plays a significant role in creating an immunosuppressive tumor microenvironment (74). A key mechanism involves the upregulation of the immune checkpoint protein PD-L1 on tumor cells. This process is intentional; studies have shown that MYC collaborates with the Kinase CDK5 to stabilize PD-L1 mRNA, thereby ensuring its elevated expression (75). This strategic upregulation of PD-L1 provides insight into a perplexing characteristic of the Group 3 microenvironment: the presence of tumor-infiltrating cytotoxic CD8+ T cells that are functionally impaired. These T cells exhibit an “exhausted” phenotype, unable to mount an effective immune response despite their proximity to tumor cells. The tumor cells, shielded by their PD-L1 expression, effectively evade the immune system’s efforts (76, 77). Consequently, the Group 3 TME exemplifies a paradigm of compromised immunity, wherein the necessary immune components are present but rendered ineffective.

In contrast to Group 3, Group 4 MB generally demonstrates a less aggressive clinical progression. The most consistent genetic hallmark of Group 4 is the presence of an isochromosome 17q (i17q); however, the specific oncogenes responsible for driving these tumors have not yet been identified (5, 78). Immunologically, Group 4 are characterized by a distinctive pattern of innate and adaptive immune cell infiltration. Notably, these tumors often exhibit a significant presence of natural killer (NK) cells, which are components of the innate immune system capable of recognizing and eliminating stressed cells without prior sensitization (5, 79, 80). Of particular interest is the observed correlation between B cell infiltration and a more favorable prognosis (44, 81). The presence of B cells, which serve as antigen-presenting cells and antibody producers, suggests the potential involvement of a broader, more coordinated immune response. Nonetheless, the precise role of these B cells, as to whether they function as active anti-tumor effectors or merely serve as passive indicators of a less immunosuppressive environment, remains an open question (82, 83). This B cell signature provides a valuable prognostic indicator and represents a potential therapeutic target.

Both Group 3 and Group 4 subtypes face a key issue: low immunogenicity. Their low mutation rates result in fewer neoantigens, reducing immune system alerts. Combined with often downregulated MHC-I machinery, these tumors effectively evade immune detection. This “coldness” limits the success of standard immune checkpoint inhibitors, highlighting the need for advanced strategies like oncolytic viruses or combination therapies to boost antigen presentation and inflame these TMEs.

## 4 The expanding frontier of immunotherapy in medulloblastoma

### 4.1 Immune checkpoint inhibitors

Immune checkpoint inhibitors (ICI) have achieved breakthrough advances in the treatment of various malignant tumors (84–86). However, their clinical application in MB still faces significant challenges (87). Current research indicates that PD-1/PD-L1 inhibitors demonstrate limited overall efficacy in MB, yet this limitation conceals subtype-specific responses and novel therapeutic opportunities.

#### 4.1.1 Limited efficacy of PD-1/PD-L1 inhibitors and underlying mechanisms

The restricted efficacy of PD-1/PD-L1 inhibitors in MB is primarily attributed to low target expression levels and tumor heterogeneity. Multiple studies have confirmed that MB generally exhibits a low tumor mutational burden and low PD-L1 expression (46, 88). Transcriptomic analyses by Bockmayr et al. revealed that the expression levels of immune-related genes in MB are among the lowest of all brain tumors, and PD-L1 protein is undetectable in most samples (17). This “immune-quiet” phenotype may be related to the genomic stability and low antigen-presenting capacity of MB. Furthermore, significant heterogeneity exists among molecular subtypes: PD-L1 expression is lowest in WNT MB, while SHH and Group 3 subtypes show detectable expression in some cases, albeit at levels significantly lower than those in other cancers (17, 89). This high degree of heterogeneity makes patient selection an unavoidable challenge in clinical applications.

The presence of the blood-brain barrier further restricts drug delivery and T-cell infiltration (90, 91). The immune microenvironment of MB is characterized by low lymphocyte infiltration. Particularly in some subtypes, downregulation of MHC class I molecules enables tumor cells to evade T-cell recognition (92), resulting in an “immune-excluded” status.

#### 4.1.2 Therapeutic potential in specific subtypes

Despite numerous challenges, research indicates that certain MB subgroups may exhibit potential sensitivity to immune checkpoint inhibitors (ICI). For instance, Group 3 MB, characterized by MYC amplification (36), although associated with a very poor prognosis, displays certain immune-activated features. Pham et al.’s research demonstrated that in mouse models of this subtype, anti-PD-1 treatment significantly enhanced the infiltration of cytotoxic T cells into tumors and extended survival (68), suggesting that the immune microenvironment may exhibit characteristics of a “hot tumor.” Furthermore, the high cellular proliferative activity and genetic instability observed in Group 3 tumors (93) may facilitate neo-antigen generation, thereby providing a basis for immune recognition. Consequently, precise molecular subtyping could enable the use of ICI in patients with specific genetic backgrounds within Group 3, such as MYC amplification or GFI1 activation, potentially overcoming current therapeutic limitations.

Given the inherent limitations of targeting the PD-1/PD-L1 axis in MB, research efforts have expanded to explore alternative immune checkpoints with more favorable expression profiles and mechanisms of action within the CNS tumor microenvironment. This has led to the investigation of several promising candidates, notably B7-H3 and CD47, which are emerging as critical mediators of immune evasion in MB.

#### 4.1.3 Emerging immune checkpoints: B7-H3, CD47

B7-H3 (CD276), a key transmembrane protein in the B7 family, is widely overexpressed in various cancers and functions as a critical immune checkpoint that facilitates tumor immune escape (94, 95). Unlike the PD-1/PD-L1 axis, which shows limited and subtype-dependent efficacy in medulloblastoma, B7-H3 exhibits broad expression and multifaceted pro-tumorigenic roles. It promotes tumor angiogenesis, invasion, and metastasis, while also suppressing T cell-mediated immunity by inhibiting cytotoxic function and cytokine production (96–99). Although its receptor remains incompletely defined, several candidate binding partners, including TLT-2, IL20RA, and AAMP, have been implicated in mediating its immunosuppressive effects (100–102). Therapeutic targeting of B7-H3 using monoclonal antibodies and CAR-T cells is under active clinical investigation.

CD47 has emerged as a master regulator of innate immune evasion. It is highly expressed on many aggressive pediatric brain tumors, including Group 3 medulloblastoma, AT/RT, and DIPG (103, 104). By engaging SIRPα on macrophages, it delivers a “don’t eat me” signal that blocks phagocytosis (105). Preclinical studies demonstrate that anti-CD47 antibodies, such as Hu5F9-G4, promote macrophage-mediated tumor cell phagocytosis, suppress cancer stem cells, and inhibit tumor growth without significant neurotoxicity (106, 107). Moreover, CD47 blockade synergizes effectively with other agents—for instance, HDAC inhibitors like CI-994, which enhance “eat me” signals and pro-inflammatory cytokine release (104). Such combinations reverse immunosuppressive cues and improve phagocytic function, leading to sustained survival benefits *in vivo*.

Together, B7-H3 and CD47 represent promising immune checkpoints with distinct mechanisms of action. Both are being leveraged in novel mono- and combination therapies aimed at overcoming immune resistance in high-risk malignancies including medulloblastoma.

### 4.2 Chimeric antigen receptor T-cell therapy

Chimeric antigen receptor T-cell (CAR-T) therapy has achieved remarkable success in the treatment of hematological malignancies (108). In recent years, its application in MB has been increasingly explored (109). As MB is a solid tumor located within the immunologically privileged central nervous system, CAR-T therapy faces multiple challenges, including tumor heterogeneity, target antigen selection, and the immunosuppressive tumor microenvironment. However, by optimizing target selection and adopting localized delivery strategies, this field is demonstrating promising therapeutic potential.

Several antigens highly expressed in MB and associated with tumorigenesis and progression have been identified, providing potential targets for CAR-T therapy. B7-H3 is widely overexpressed in more than 80% of MB samples, particularly in Group 3 and Group 4 subtypes (110). It facilitates tumor immune evasion and promotes cell proliferation and metastasis (111, 112), making it a highly attractive target. Preclinical studies have shown that B7-H3-targeted CAR-T cells can effectively suppress tumor growth (113); HER2 exhibits variable expression across medulloblastoma (MB) subtypes, with notable overexpression in certain subgroups such as SHH and Group 3. Preclinical studies have demonstrated that HER2-directed CAR-T cells can induce tumor regression (114–117). GD2, another promising target, is highly and specifically expressed in SHH-MB, making it a suitable subtype-specific therapeutic candidate. GD2-targeted CAR-T therapy has already shown applicability in other neuroectodermal tumors (118, 119). In MB, IL13Rα2 and EPHA2 are highly expressed and associated with an aggressive phenotype (120, 121). Donovan et al. (115) confirmed that intrathecally delivered CAR-T cells targeting these antigens elicit significant tumor regression in aggressive Group 3 MB mouse models.

Traditional intravenous infusion of CAR-T cells for brain tumors faces limitations such as inefficient migration of cells to intracranial tumor sites and systemic toxicities like cytokine release syndrome (CRS) (122, 123). Recent breakthroughs indicate that local delivery methods can directly bypass the blood-brain barrier, significantly increasing T-cell concentration at the tumor site. Studies by Donovan et al. demonstrated in Group 3 MB animal models that compared to intravenous infusion, intrathecal delivery of CAR-T cells targeting EPHA2, HER2, or IL13Rα2 not only markedly enhanced antitumor activity and animal survival but also substantially reduced the risk of systemic CRS and neurotoxicity (115). This approach provides a novel strategy for achieving highly efficient and low-toxicity immunotherapy for MB.

Despite these promising advances, several critical limitations must be addressed before CAR-T therapy can be widely translated into clinical practice for MB. Antigen heterogeneity remains a major obstacle, as MB subtypes exhibit variable expression of target antigens, and even within a single tumor, expression can be patchy and heterogeneous (100). This heterogeneity creates selection pressure that may lead to antigen escape, where tumor cells downregulate or lose the targeted antigen to evade CAR-T recognition, resulting in tumor relapse. Multi-targeted CAR-T designs or sequential combination therapies are being explored to mitigate this risk.

Beyond target-related challenges, neurotoxicity poses a significant concern. Unlike hematologic malignancies, MB resides within the delicate and immunologically specialized central nervous system. CAR-T cell activation within the confined intracranial space can trigger severe adverse events, including cytokine release syndrome (CRS) and immune effector cell-associated neurotoxicity syndrome (ICANS) (112, 113). While local delivery routes such as intrathecal or intraventricular injections reduce systemic toxicity, they may concentrate inflammatory responses within the CNS, potentially causing cerebral edema, neuronal damage, or fatal neuroinflammation. Preclinical models have reported such toxicities, underscoring the need for careful dose optimization and incorporation of safety switches that allow rapid elimination of CAR-T cells if severe toxicity occurs (105).

Compounding these biological hurdles, pediatric-specific safety considerations are paramount. Children, particularly infants and young children, have developed nervous systems that may be more vulnerable to therapy-induced neurotoxicity. Long-term sequelae, including cognitive impairment, endocrine dysfunction, and secondary malignancies, are of particular concern in this population. The risk-benefit calculus differs substantially from adult patients, necessitating age-appropriate toxicity monitoring, long-term follow-up protocols, and potentially modified treatment regimens for pediatric MB patients.

Finally, manufacturing and logistical challenges remain substantial. Autologous CAR-T cell production is time-consuming and resource-intensive, which may be impractical for patients with rapidly progressive disease. Tumor-induced T-cell dysfunction in heavily pretreated patients can also impair the quality of harvested T cells, reducing manufacturing success rates and therapeutic efficacy. Allogeneic “off-the-shelf” CAR-T products are being explored to address these limitations, though they carry risks of graft-versus-host disease and rejection. Addressing these challenges will require iterative refinement of CAR-T design, rigorous preclinical safety testing in pediatric models, and carefully designed early-phase clinical trials with robust monitoring for both efficacy and toxicity.

### 4.3 Other modalities

Beyond immune checkpoint inhibitors and CAR-T cell therapy, a variety of novel immunotherapeutic strategies offer diversified directions for the treatment of MB. These approaches aim to activate anti-tumor immune responses through different mechanisms or reverse the immunosuppressive state of the tumor microenvironment (TME).

#### 4.3.1 Oncolytic virotherapy

Oncolytic viruses (such as herpes simplex virus G207 and measles virus MV-NIS) not only selectively infect and lyse tumor cells (124–126), more importantly, can reshape an immunologically “cold” TME into a “hot” one. The process of viral replication and tumor cell lysis releases tumor-associated antigens and danger signal molecules, thereby promoting dendritic cell (DC) maturation, T cell activation and infiltration, and triggering a systemic anti-tumor immune response (127). Several clinical trials (e.g., NCT03911388, NCT02962167) are currently evaluating the safety and efficacy of oncolytic viruses in recurrent/refractory MB.

#### 4.3.2 Cancer vaccine strategies

Vaccine therapies induce specific anti-tumor responses through active immunization. Among them, dendritic cell (DC) vaccines are loaded with tumor antigens (such as tumor lysates or specific peptides) to effectively present antigens and activate T cells (128). Peptide vaccines, on the other hand, utilize the direct immunogenicity of MB-associated antigens (63, 129). Although early-stage clinical trials have been small in scale and face challenges such as antigen presentation efficiency, the combination of vaccines with immune adjuvants or other immunotherapies remains a key direction for enhancing immune memory and treatment depth.

#### 4.3.3 NK cell therapy

Natural killer (NK) cells have therapeutic potential due to their MHC-unrestricted killing ability. However, their function in the MB TME is often compromised by immunosuppressive factors such as TGF-β (130). To improve efficacy, current strategies focus on genetic engineering: for example, introducing a dominant-negative TGF-β receptor II (DNRII) to resist inhibitory signals, or engineering NK cells to express chimeric antigen receptors (CARs) targeting MB-associated antigens to enhance tumor recognition and cytotoxicity (131). Local delivery methods, such as intrathecal infusion, also help increase the localized concentration of NK cells at the tumor site.

#### 4.3.4 Targeting tumor-associated macrophages

TAMs are highly abundant in the SHH subtype, where they predominantly exhibit an M2-polarized phenotype that fosters immune evasion and tumor progression (132). Therapeutic strategies targeting TAMs include: CSF1R inhibitors (133), which disrupt the survival and recruitment of TAMs. Preclinical studies demonstrate synergistic antitumor effects when combined with radiotherapy or chemotherapy; CD47–signal regulatory protein alpha (SIRPα) blockers (103), which interfere with the “don’t eat me” signal and promote macrophage phagocytosis of tumor cells. Although evidence in MB remains preliminary, promising results in other cancers support their translational potential.

These approaches are mutually complementary and may be integrated with conventional radiotherapy, chemotherapy, immune checkpoint inhibitors, or cellular therapies to address the immunosuppressive and heterogeneous nature of MB.

## 5 Clinical translation, challenges and future perspectives

**Figure 5:**
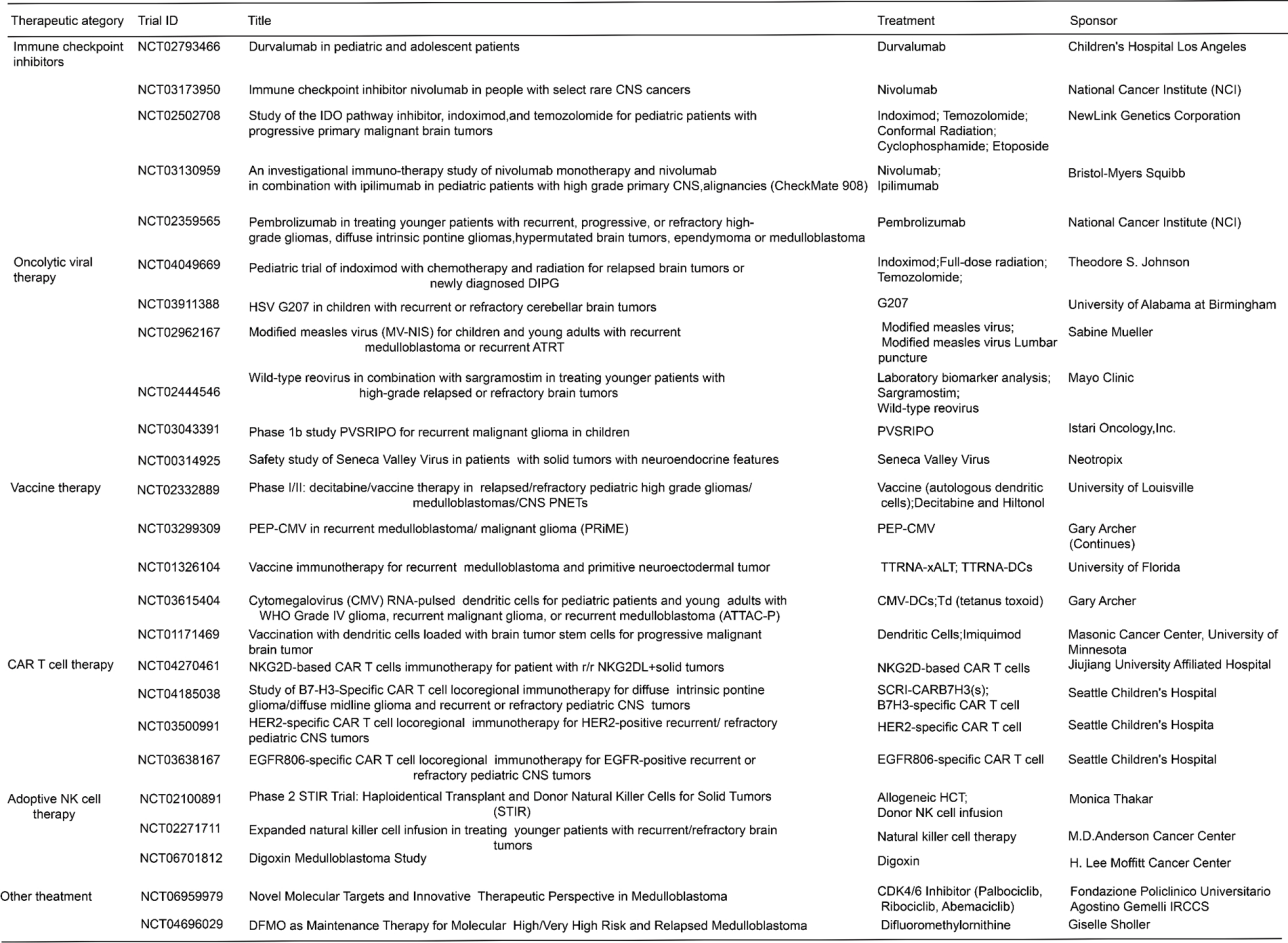
Overview of current clinical trials investigating immunotherapeutic strategies for medulloblastoma. The figure summarizes ongoing or completed clinical trials categorized by therapeutic approach, including immune checkpoint inhibitors, oncolytic viral therapy, vaccine therapy, CAR T-cell therapy, adoptive NK cell therapy, and other novel treatments. Key agents under evaluation are listed with their respective trial identifiers and sponsors, reflecting the diverse landscape of immunotherapy development aimed at overcoming the immunosuppressive tumor microenvironment in medulloblastoma.

Despite demonstrating preclinical promises in MB, the clinical translation of immunotherapies remains challenging, Current clinical trials investigating immunotherapy for medulloblastoma are summarized in **Figure 5**. A major barrier is the blood-brain barrier (BBB), which severely restricts delivery of therapeutic agents, including monoclonal antibodies, cytokines, and CAR-T cells, to tumor sites. Even local delivery routes such as intrathecal or intraventricular injections can lead to suboptimal drug distribution and penetration. Furthermore, the immunosuppressive TME in MB, enriched with TAMs, regulatory T cells (Tregs), TGF-β, and IL-10, promotes T cell exhaustion, functional suppression, and antigen escape.

Immunotherapies such as immune checkpoint inhibitors (ICIs) and CAR-T cells can also induce significant immune-related adverse events (irAEs), including cytokine release syndrome (CRS) and immune effector cell-associated neurotoxicity syndrome (ICANS). These toxicities are especially concerning pediatric patients due to developing organs and distinct tolerance profiles. Moreover, reliable predictive biomarkers are currently lacking. Conventional markers like PD-L1 expression and tumor mutational burden (TMB) are generally low and heterogeneous in MB, underscoring the need for novel biomarkers, such as immune infiltration signatures, serum cytokine profiles, or radiomic features—to guide patient stratification.

Despite these challenges, future directions remain promising. Key priorities include: developing more physiologically relevant models; designing rational combination therapies; applying spatial transcriptomics and multiplex immunofluorescence to map immune “cold” and “hot” zones; and exploring indirect TME modulation via targets such as TAAs or vascular normalization to enhance immune cell infiltration and function.

## 6 Conclusion

The heterogeneity of the immune microenvironment in medulloblastoma should be viewed not as a barrier, but as an opportunity for precision immunotherapy. Through molecular stratification of patients, development of novel immune targets and innovative delivery techniques, immunotherapy holds great promise for bringing substantial clinical benefits to high-risk and relapsed/refractory MB patients. Future success will depend on interdisciplinary collaboration and efficient translation from bench to bedside.
